# Significant diversity of human anelloviruses revealed by novel viral sequences identified in human metagenomic data

**DOI:** 10.1099/jgv.0.002199

**Published:** 2025-12-22

**Authors:** Shaokun Pan, Xingyue Zhao, Qi Shi, Zhongliang Shen, Jing Liu, Wang Li, Youhua Xie

**Affiliations:** 1 Shanghai Institute of Infectious Diseases and Biosecurity, Key Laboratory of Medical Molecular Virology (MOE/NHC/CAMS), Shanghai Frontiers Science Center of Pathogenic Microbes and Infection, Department of Microbiology and Parasitology, School of Basic Medical Sciences, Shanghai Medical College, Fudan University, Shanghai, PR China; 2 Clinical Medical College of Anhui Medical University, Anhui, PR China; 3 Department of Infectious Diseases, Shanghai Institute of Infectious Diseases and Biosecurity, Shanghai Key Laboratory of Infectious Diseases and Biosafety Emergency Response, National Medical Center for Infectious Diseases, Huashan Hospital, Fudan University, Shanghai, PR China; 4 Key Laboratory of Medical Epigenetics and Metabolism, Institutes of Biomedical Sciences, School of Basic Medical Sciences, Shanghai Medical College, Fudan University, Shanghai, PR China; 5 The Affiliated Taizhou People’s Hospital of Nanjing Medical University, Taizhou, Jiangsu 225300, PR China; 6 Department of Laboratory Medicine, School of Medicine, Jiangsu University, Zhenjiang, PR China

**Keywords:** anellovirus, *in silico *screening, metagenomics, torque teno midi virus (TTMDV), torque teno mini virus (TTMV), torque teno virus (TTV)

## Abstract

Human torque teno viruses are emerging infectious agents distributed globally and have increasingly been reported to be associated with human diseases. To identify potential anelloviral sequences in available metagenomic data, an *in silico* screening was performed mainly employing the ORF1, ORF2 and ORF3 nucleotide/protein queries of known human anelloviruses and identified 217 complete ORF1 regions. Pairwise nucleotide‐identity analysis with a 69% cut‐off – consistent with ICTV species demarcation – revealed 117 novel species across the 3 major human‐infecting genera: 15 in *Alphatorquevirus*, 51 in *Betatorquevirus* and 51 in *Gammatorquevirus*. In nearly all cases, these species assignments correspond precisely to monophyletic clusters in maximum‐likelihood phylogenies of ORF1 amino acid sequences. Using AlphaFold3-guided modelling together with representative ORF1 alignments, we delineated capsid motifs – the conserved jelly-roll (JR) *β*-sandwich core (*β*-strands B–I) and the outward projection domains P1/P2 – and quantified motif lengths across genera, revealing tightly constrained JR lengths with genus-specific but overlapping variation in P1/P2. A few exceptions – where pairwise‐based groupings split or merge slightly differently – highlight ongoing challenges in delineating rapidly evolving viruses. Notably, the two deeply branching isolates retain the canonical JR core while exhibiting a TTMDV-like short P2, indicating preservation of key capsid architecture in the newly proposed genus. This work nearly doubles the known species richness of human anelloviruses and introduces a novel genus, underscoring the vast, hidden diversity of the gut virome and its potential impact on human health. By coupling taxonomy with structure-informed ORF1 motif analysis, our study provides biological context for these lineages and a framework for future functional and immunological investigations.

## Data Summary

All assembled anellovirus genomes and ORF1 sequences generated in this study have been deposited in a single figshare submission titled ‘Significant diversity of human anelloviruses revealed by novel viral sequences identified in human metagenomic data’ (File S1, available in the online Supplementary Material, FASTA; plus supplementary figures/tables). The dataset is publicly available at Figshare DOI: https://doi.org/10.6084/m9.figshare.30070036 and is cited in the References [1]. Reference sequences analysed in this study are available from GenBank; accession numbers are listed in Table S5. Upon acceptance, we will submit the newly assembled sequences to GenBank as standard records and add accession numbers to the manuscript.

AlphaFold3 structural models (capsid ORF1). Representative capsid proteins – HTTVa_002 (TTV), HTTVb_001 (TTMV), HTTVg_002 (TTMDV) and HTTVgl_001 (proposed new genus) – were modelled with AlphaFold3 via the AlphaFold Server (alphafoldserver.com). For each protein, we provide the top-ranked model (model_0.cif, CIF/PDBx-mmCIF), alongside its confidence summary (*_summary_confidences_0.json) and Predicted Aligned Error (PAE) output (if available). Where applicable, we also include the full-data JSON for the top-ranked model (*_full_data_0.json). The authoritative ORF1 FASTA sequences and a sanitized AlphaFold input record (*_job_request_public.json) are deposited to support reproducibility; if any discrepancy arises, the FASTA takes precedence. All structural figures and JR/P1/P2 length quantification use model_0.cif. (Note: the AlphaFold Server generates five candidate models per sequence; to reduce redundancy and file size, only the top-ranked model is deposited. Additional models can be provided upon request).

## Introduction

Human torque teno viruses (HTTVs), firstly discovered in 1997, are emerging infectious agents distributed globally. The family *Anelloviridae* comprised more than 30 genera [2], and HTTVs are comprised of 3 main genera of *Alphatorquevirus* [torque teno virus (TTV)], *Betatorquevirus* [torque teno mini virus (TTMV)] and *Gammatorquevirus* [torque teno midi virus (TTMDV)] and recently assigned genera of *Hetorquevirus* [3], *Yodtorquevirus*, *Lamedtorquevirus*, *Memtorquevirus* and *Samektorquevirus* [4]. *Anelloviridae* also includes hundreds of species that infect animals including chimpanzees [5], tupaias [6], pigs [7], chickens, cows [8], cats and dogs [9], rodents and bats [10], boar [11] and camels [12]. Anelloviruses are non-enveloped, negative-sense single-stranded circular DNA viruses with genomes ranging in size from 1.6 to 3.9 kb [10]. Their genome can be divided into a UTR and a coding region. UTR is relatively conserved, characterized by a GC-rich region of about 100 nt. The coding region contains a large ORF1 covering almost 70% of the genome [13]. Accumulating structural and comparative evidence indicates that ORF1 encodes the principal capsid protein adopting a single jelly-roll (SJR) *β*-sandwich core with outward projection modules. In particular, insertions within the jelly-roll (JR) – often between *β*-strands H and I – form surface-exposed projections that vary across genera, whereas the core *β*-strands (B–I) remain conserved in topology [14]. The genome size varies among different isolates, ranging from 3.6 to 3.9 kb for TTV, 2.8 to 3.0 kb for TTMV and 3.24 to 3.25 kb for TTMDV [10,13].

HTTV DNA in human samples has been detected all over the world [13,15, 16], and the rate of virus prevalence varies greatly among different populations [10,17,21]. HTTVs have been detected in various samples including serum [13,22], saliva, cervical secretion, faeces, throat swabs, liver, bile, gastric tissue, semen, hair and skin, bone marrow, lymph nodes, muscles, umbilical cord blood, thyroid, lungs, spleen, pancreas, kidney, cerebrospinal fluid, nervous tissue and peripheral blood mononuclear cells [10]. It is uncertain whether the infection of HTTVs causes any human disease, though an accumulating body of reports has associated HTTVs with hepatitis [22], respiratory diseases [23], cancer [24], haematological disorders [25], autoimmune disorders [26,27], Hodgkin’s lymphoma [13], periodontitis [16], melanoma [28] and acute promyelocytic leukaemia [29]. Notably, the apparent disconnect between high prevalence and unclear disease association motivates analyses that go beyond sequence description, including structure-informed comparisons of ORF1 motifs that may relate to host interaction.

Metagenomics was first proposed in 1998 to study genetic materials recovered directly from environmental samples [30]. Because of its ability to reveal the previously hidden diversity of bacterial microbiome and viral microbiome (virome), metagenomics offers a powerful tool for discovering new microbial species. Using metagenomics, we had previously identified 2 TTMV species (TTMV 26, isolate SHA, KY462770; TTMV 32, isolate 222, KU041847) in gingival tissue from periodontitis patients and in serum samples from Hodgkin’s lymphoma patients [13,15, 16] and one isolate (TTMV-204, KU243129) belonging to TTMV 8 [15]. With the rapid development of high-throughput sequencing technology, a large number of raw genomic and metagenomic data have been generated and deposited in the public databases such as GenBank.

In the present study, we performed an *in silico* screening for potential anellovirus sequences in publicly available metagenomes. A total of 217 new HTTV isolates containing intact ORF1 were identified in a human gut metagenome. According to the current ICTV species demarcation criteria based on ORF1 pairwise nucleotide identity of 69% [2], 117 novel species in 3 established genera of *Anelloviridae* were identified, with 15 novel species in *Alphatorquevirus*, 51 novel species in *Betatorquevirus* and 51 novel species in *Gammatorquevirus*, respectively. Additionally, one novel genus with two novel species clustered closely together with known HTTVs was also identified. Beyond cataloguing diversity, we integrated reference sequences into ORF1/genome-length comparisons and used AlphaFold3 with representative ORF1 alignments to delineate conserved capsid motifs – JR *β*-strands B–I and the projection domains P1/P2 – across *Alphatorquevirus*, *Betatorquevirus*, *Gammatorquevirus* and the newly proposed genus. These analyses show that the new isolates retain the canonical capsid core while exhibiting genus-typical variation in the projections, providing structural/functional context for the phylogenetic placements reported here.

## Methods

### *In silico* screening for new HTTV sequences

*In silico* screening of potential anelloviral sequences in available metagenomes (http://www.ncbi.nlm.nih.gov/sites/genome) was performed using tblastx and the ORF1, ORF2 and ORF3 protein sequences from all representative species of *Alphatorquevirus*, *Betatorquevirus*, *Gammatorquevirus* and *Hetorquevirus* as queries, and a cutoff of 35% sequence identity was employed.

### Phylogenetic analysis

Complete ORF1 amino acid sequences (new isolates and representative references) were first aligned with MAFFT v7.525 using the --auto setting [31]. The resulting alignment was then trimmed in TrimAL v1.4 [32] with the -gappyout option to remove poorly aligned or gap‐rich columns. A maximum‐likelihood phylogeny was inferred from the trimmed alignment using IQ‐TREE v2.4.0 [33], specifying the LG+F+G4 substitution model and performing 1,000 Shimodaira–Hasegawa approximate likelihood ratio test (SH‐aLRT) and 1,000 ultrafast bootstrap replicates to assess node support. The final tree was visualized in R with the ggtree package [34], and clades were annotated according to genera.

### Sequence identity analysis and novel species assignment

All complete ORF1 nucleotide sequences (newly identified isolates together with representative reference sequences) were first extracted and compiled into a single FASTA file. Pairwise identity values were calculated using the Sequence Demarcation Tool (SDT) v1.2 [35] with default gap‐treatment parameters. Briefly, SDT performed a full global alignment for each pair of ORF1 sequences and then computed nucleotide identity as the percentage of identical sites over the alignment length, excluding terminal overhang gaps. Following current ICTV guidelines for *Anelloviridae*, a 69% pairwise nucleotide‐identity threshold was adopted to demarcate species within each genus [2]. Any pair of isolates (or isolate–reference pair) with ORF1 identity ≥69% was considered to belong to the same species; conversely, pairs with identity <69% were deemed to represent different species. New isolates that failed to exceed 69% identity to any existing reference were marked as candidate novel species. Among these candidates, isolates sharing ≥69% identity with one another but <69% identity to all other isolates or references were clustered into the same novel species group. Isolates that remained singleton (no partner ≥69% identity) were each assigned to their own novel species. This procedure yielded a set of novel species assignments for the ORF1 dataset. Before ICTV formally approves new species names, we assigned provisional labels (e.g. ‘TTVx1’ for novel *Alphatorquevirus* lineages, ‘TTMVx1’ for novel *Betatorquevirus* lineages and ‘TTMDVx1’ for novel *Gammatorquevirus* lineages). These temporary identifiers prevent confusion that would arise if we continued ICTV’s existing numbering (for example, a newly detected Alphatorquevirus species might later be designated ‘*Alphatorquevirus homin32*’ or abbreviated ‘TTV32’). Once ICTV publishes the official nomenclature, our provisional labels can be unambiguously mapped to the formal names.

### AlphaFold3 structure prediction of ORF1 (capsid)

Representative ORF1 amino acid sequences – HTTVa_002 (*Alphatorquevirus*), HTTVb_001 (*Betatorquevirus*), HTTVg_002 (*Gammatorquevirus*) and HTTVgl_001 [proposed new genus (NG)] – were modelled with AlphaFold3 via the AlphaFold Server in monomer mode using default settings [36]. For each sequence, the AlphaFold server returned five candidate models; unless otherwise stated, figures and region-length quantification used the top-ranked model (model_0.cif) selected by highest mean the predicted Local Distance Difference Test (pLDDT) and lowest PAE. Only model_0.cif and its confidence outputs are deposited (see Data Summary). Consistent with prior reports for anellovirus ORF1, low-complexity/disordered N-terminal segments often showed low confidence (pLDDT <50) and were retained for context in figures but excluded from region-length statistics and across-model comparisons.

### ORF1 alignment for structural annotation (representative sequences)

To support structural/motif annotation rather than tree inference, we generated a representative capsid (ORF1) alignment comprising three reference species TTV21 (GenBank accession no. AF348409), TTMV15 (JX134044), TTMDV3 (EF538875) and newly identified isolates (HTTVa_002, HTTVb_001, HTTVg_002, HTTVgl_001 and HTTVgl_002). Sequences were aligned with MAFFT v7.525 using L-INS-i and default gap penalties [31]. Poorly aligned low-complexity segments at the extreme N-termini were masked for visualization and excluded from boundary calls. This structural alignment was used solely to corroborate JR/P1/P2 boundaries (in conjunction with AlphaFold3 models) and to guide region selection for structural superposition.

### Definition and quantification of JR, P1 and P2 regions

Annotation of the JR *β*-sandwich core and the two surface projections (P1 and P2) combined structure-based features from AlphaFold3 models with sequence evidence from multiple-sequence alignment (MSA) of representative new isolates and reference strains. (1) Structure anchor: putative *β*-sandwich strands forming the compact capsid core were identified by secondary-structure assignment on the AlphaFold3 models and by local pLDDT support (typically ≥70). (2) Sequence support: conserved sequence blocks corresponding to the core *β*-strands were located in the MSA; genus-specific loop insertions were mapped to P1/P2 when they projected from the JR core in the models. (3) Boundary calling: JR/P1/P2 boundaries were set at transitions where secondary-structure continuity and topology relative to the JR core were concordant with MSA conservation. Ambiguous or low-confidence residues (pLDDT <50) at region edges were not assigned. For each model, region length was recorded as the number of residues assigned to JR**,** P1 and P2, respectively.

### Structural superposition and visualization

To compare capsid architecture across genera, AlphaFold3 models of HTTVa_002, HTTVb_001, HTTVg_002 and HTTVgl_001 were superposed in PyMOL v3.0.0. Unless otherwise stated, superpositions and region-length quantification used the top-ranked model (model_0.CIF) for each protein, selected by highest mean pLDDT and lowest PAE. We first aligned the JR core residues (as defined above) using structure-based alignment (PyMOL cealign on core selections) to minimize bias from variable loops and then refined with super where appropriate. Superposed models were displayed as full-length ORF1 with JR/P1/P2 highlighted according to assigned boundaries and coloured consistently across panels. All five models (model_0–model_4.CIF) per protein, confidence outputs for the top-ranked model and a file-level README are available in the Figshare item cited in the Data Summary.

## Results

### Human anellovirus sequences found in human metagenomes

We employed known HTTV (all represent species from the genus of *Alphatorquevirus*, *Betatorquevirus*, *Gammatorquevirus* and *Hetorquevirus*) ORF1 amino acid sequences as queries (cutoff: 35% sequence identity) to screen 13 human metagenome projects and obtained multiple matches (Table S1). Twelve of the projects contain contigs harbouring short ORF1 matches (less than 500 bp). The remaining project that deals with the gut metagenome from Malawi infants with severe acute malnutrition (WGS Project: CYGL) includes contigs with long ORF1 matches (more than 1.8 kb) [37]. Given that all known HTTV ORF1 genes exceed 1,887 bp, we assumed that only the contigs with the long matches might harbour an intact or near full-length ORF1 gene. Thus, the metagenome from project CYGL was subject to further analysis.

The best matches were obtained with the ORF1 amino acid sequences of TTV 11 (GenBank accession no. AF345524), TTMV2 (GenBank accession no. AB038629) and TTMDV 2 (GenBank accession no. AB290919) as queries in the initial screening. Therefore, the CYGL gut metagenome was further analysed using the ORF1, ORF2 and ORF3 protein sequences of the three viruses as queries (cutoff: 35% sequence identity). The screening identified 181 TTV sequences in 175 contigs, 162 TTMV sequences in 160 contigs and 423 TTMDV sequences in 417 contigs (Fig. S1). Among them, 217 HTTV sequences containing intact ORF1 with both start and stop codons were found in 204 contigs (Table S2, Fig. S1).

### Novel genus identified in the family *Anelloviridae*

**Fig. 1.**
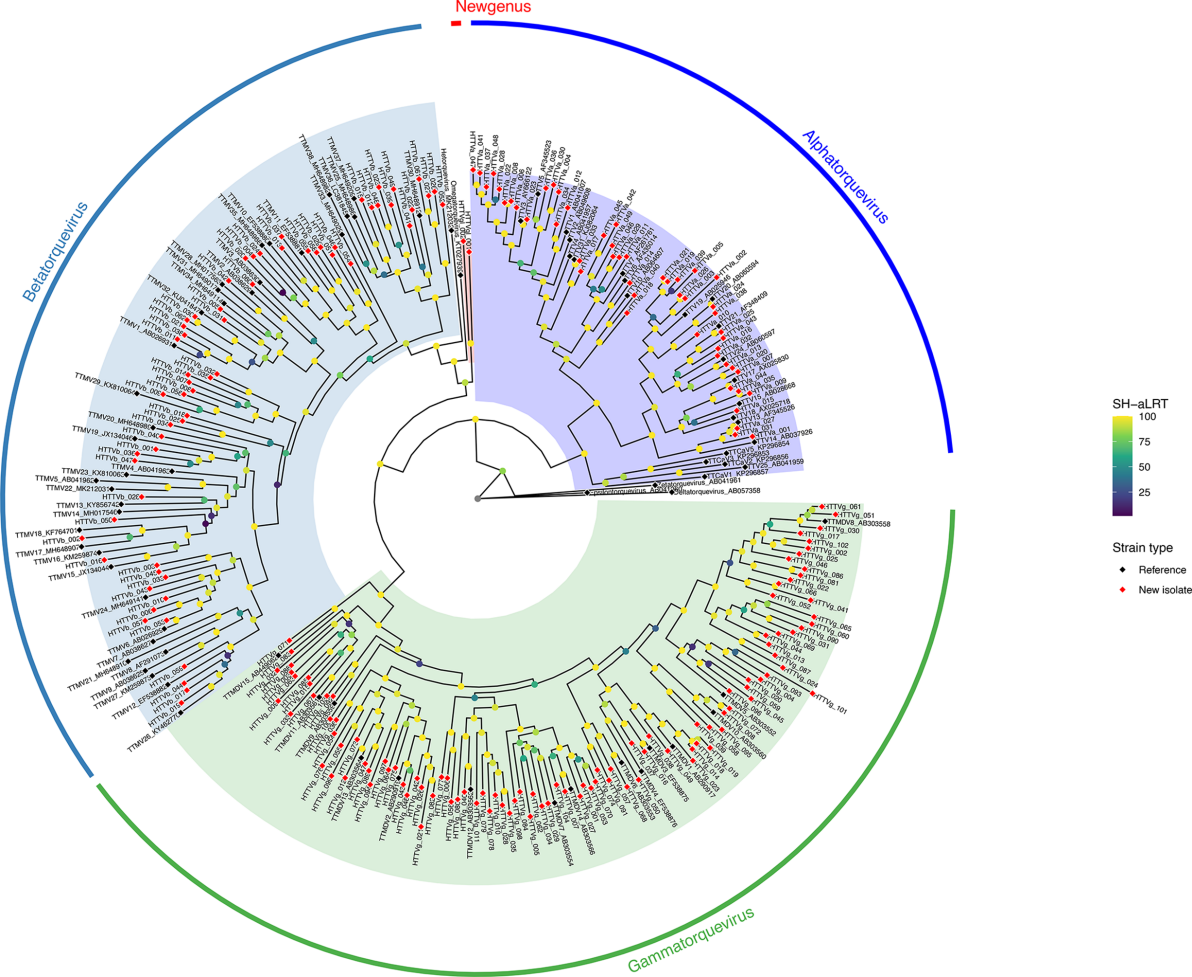
Maximum‐likelihood phylogeny of HTTV ORF1 amino acid sequences. The tree was inferred using IQ‐TREE v2 under the LG+F+G4 substitution model, with 1,000 SH-aLRT replicates and 1,000 ultrafast bootstrap replicates to assess node support. Newly identified isolates (HTTVa_001–HTTVa_049, HTTVb_001–HTTVb_062, HTTVg_001–HTTVg_104 and HTTVgl_001–HTTVgl_002) are shown alongside representative reference sequences from *Alphatorquevirus*, *Betatorquevirus*, *Gammatorquevirus*, *Hetorquevirus*, *Omegatorquevirus*, *Epsilontorquevirus* and *Zetatorquevirus*. Branch labels indicate SH-aLRT support (%) in an emerald gradient. Clades corresponding to the three major genera of human anelloviruses are highlighted: *Alphatorquevirus* (blue), *Betatorquevirus* (steel blue) and *Gammatorquevirus* (green). The two isolates HTTVgl_001 and HTTVgl_002 form a distinct branch (red) relative to *Betatorquevirus*, *Gammatorquevirus*, *Hetorquevirus* and *Omegatorquevirus* but remain within the larger clade that also contains *Alphatorquevirus*, *Epsilontorquevirus* and *Zetatorquevirus*.

A maximum‐likelihood phylogeny of the complete ORF1 amino acid sequences, including all newly identified HTTV isolates alongside established reference strains, showed robust clustering of 49 isolates (HTTVa_001–HTTVa_049) within *Alphatorquevirus*, 62 isolates (HTTVb_001–HTTVb_062) within *Betatorquevirus* and 104 isolates (HTTVg_001–HTTVg_104) within *Gammatorquevirus* (Fig. 1, Table S2).

**Fig. 2.**
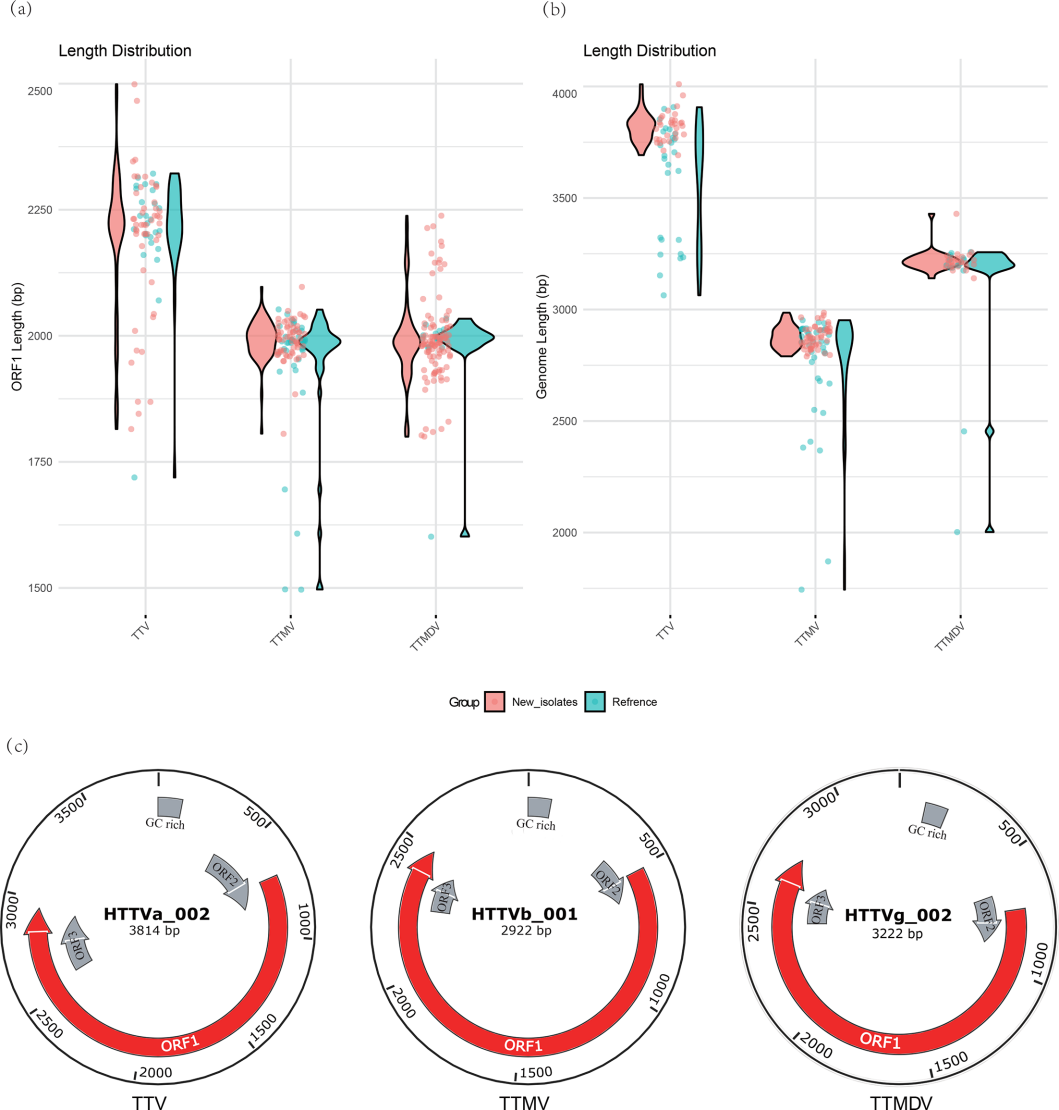
ORF1 and whole‐genome length distributions and representative genome architectures for HTTVs. (**a**) Violin plots showing the distribution of ORF1 coding‐region lengths (in base pairs) among complete ORF1 sequences for the three genera: *Alphatorquevirus* (TTV), *Betatorquevirus* (TTMV) and *Gammatorquevirus* (TTMDV). Each point represents an individual isolate, with red circles denoting newly identified sequences and blue circles denoting reference sequences. The red and blue violin shapes illustrate the overall length distributions within each genus, while the overlaid jittered points indicate the exact values for individual isolates. (**b**) Violin plots showing the distribution of complete genome lengths (in base pairs) for fully assembled isolates of TTV, TTMV and TTMDV. Red circles represent newly identified sequences and blue circles represent reference sequences. Violin shapes indicate the overall distributions, with jittered points showing individual genome lengths. (**c**) Circular genome maps for three representative isolates – HTTVa_002 (*Alphatorquevirus*, genome length=3,814 bp), HTTVb_001 (*Betatorquevirus*, genome length=2,922 bp) and HTTVg_002 (*Gammatorquevirus*, genome length=3,222 bp) – to illustrate canonical anellovirus organization. In each circle, ORF1 is shown in red, ORF2 and ORF3 in grey arrows and the GC-rich region in a shaded box. Genomic coordinates (in kilobases) are indicated around the circumference. Continuous arrows denote ORFs in the forward (+) orientation; their relative lengths and positions are proportional to nucleotide coordinates.

Within the *Alphatorquevirus* clade, the 49 HTTVa ORF1 sequences range from 1,815 to 2,499 bp (median=2,220 bp; mean=2,188.9 bp; Fig. 2a, left). Of these, 31 genomes were completely recovered: their lengths span from 3,692 to 4,010 bp (median=3,828 bp; mean=3,820 bp; Fig. 2b, left). All *Alphatorquevirus* genomes share the canonical three‐ORF architecture (ORF1, ORF2 and ORF3) plus a GC‐rich region, with ORF1 typically occupying roughly 70% of the genome (as illustrated by HTTVa_002; Fig. 2c, left). These values are broadly consistent with those of reference *Alphatorquevirus* genomes (ORF1: median=2,227 bp, mean=2,212 bp; genome: median=3,683 bp, mean=3,575 bp), supporting the similarity between newly identified and previously reported members of this genus.

In the *Betatorquevirus* group, the 62 HTTVb ORF1 sequences span 1,806 to 2,097 bp (median=1,995 bp; mean=1,992 bp; Fig. 2a, middle). Among these, 51 *Betatorquevirus* genomes were complete, ranging from 2,790 to 2,986 bp in length (median=2,873 bp; mean=2,876 bp; Fig. 2b, middle). Their genome organization likewise follows the three‐ORF plus GC‐rich region format, with ORF1 comprising ~70% of full length (illustrated by HTTVb_001; Fig. 2c, middle). Again, these parameters closely match those of reference *Betatorquevirus* sequences (ORF1: median=1,989 bp, mean=1,944 bp; genome: median=2,854 bp, mean=2,748 bp).

Within *Gammatorquevirus*, the 104 HTTVg ORF1 sequences measure 1,800 to 2,238 bp (median=1,986 bp; mean=1,993 bp; Fig. 2a, right). Twenty-five of those were recovered as complete genomes, which range from 3,140 bp to 3,429 bp (median=3,212 bp; mean=3,219.5 bp; Fig. 2b, right). As with the other genera, *Gammatorquevirus* genomes carry three ORFs plus a GC-rich region, and ORF1 spans ~70% of the genome (exemplified by HTTVg_002; Fig. 2c, right). These characteristics are in line with those of reference *Gammatorquevirus* genomes (ORF1: median=1,984 bp, mean=1,995 bp; genome: median=3,198 bp, mean=3,082 bp), again confirming the overall concordance between the new and reference datasets.

Interestingly, the two isolates HTTVgl_001 and HTTVgl_002 form a unique, well‐supported clade that is distinct from *Betatorquevirus*, *Gammatorquevirus*, *Hetorquevirus* and *Omegatorquevirus*. Despite this separation, they still fall within the broader lineage that includes *Alphatorquevirus*, *Epsilontorquevirus* and *Zetatorquevirus*. Moreover, these isolates share only 55.8% pairwise nucleotide identity across the complete ORF1, further justifying their classification as a novel genus comprising two new species (Fig. 1).

### Novel species identified in the genus *Alphatorquevirus*

**Fig. 3.**
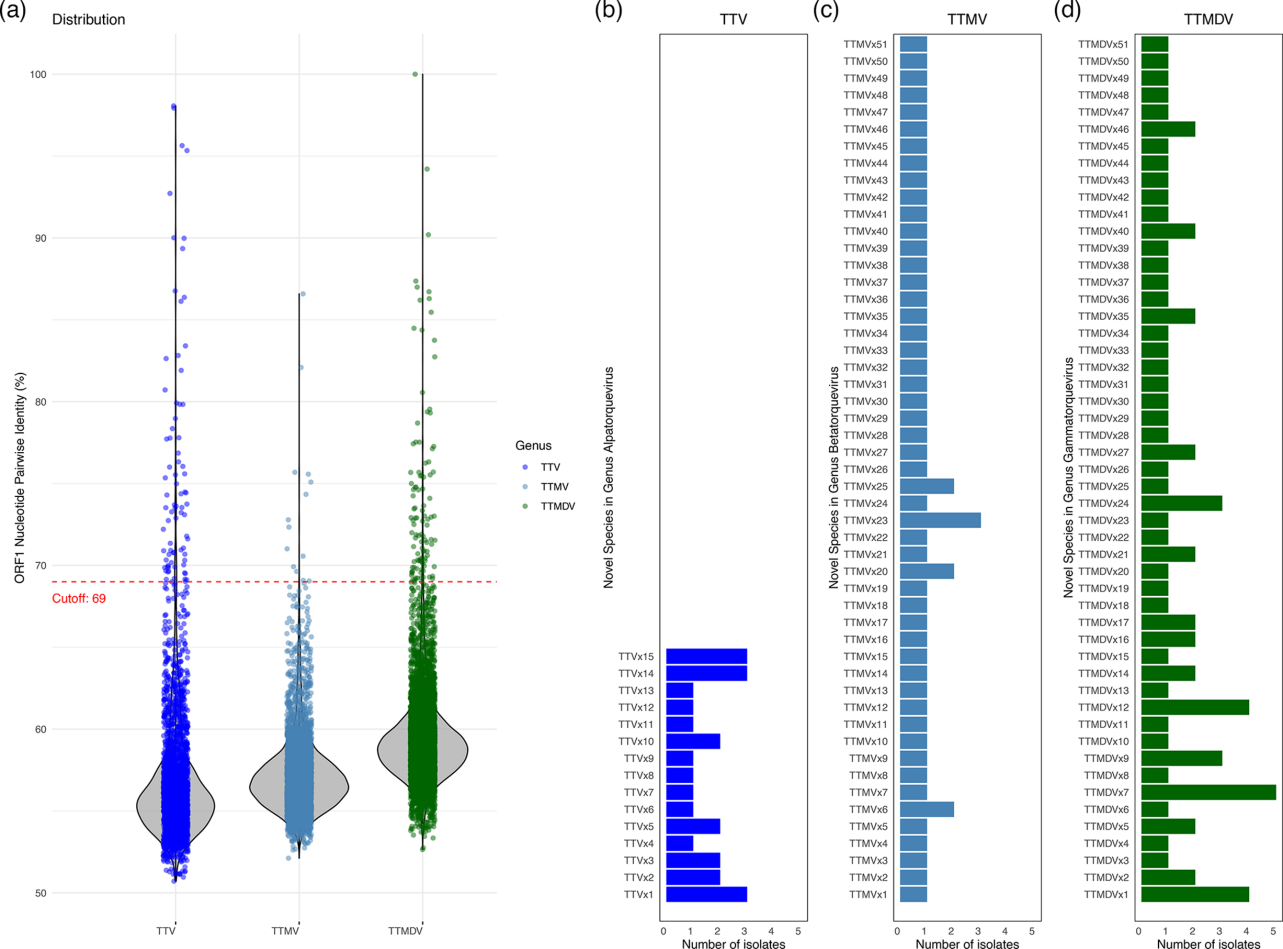
ORF1 nucleotide identity distributions and counts of novel species per genus. (**a**) Violin plots overlaid with jittered points showing pairwise ORF1 nucleotide identity values (calculated by SDT v1.2) between each newly identified isolate and representative reference sequences within the three genera. The blue points and violin represent 49 isolates plus selected reference sequences in *Alphatorquevirus*, the dark‐blue points and violin represent 62 isolates plus selected references in *Betatorquevirus* and the dark-green points and violin represent 104 isolates plus selected references in *Gammatorquevirus*. The solid red lines mark the median identity in each group, and the dashed red horizontal line indicates the 69% identity cutoff used to demarcate species. (b–d) Horizontal bar charts showing, for each genus, the newly defined species and the number of isolates assigned to each one. Panel (**b**) lists the novel *Alphatorquevirus* species (TTVx1–TTVx15) and their isolate counts; panel (**c**) shows the novel *Betatorquevirus* species (TTMVx1–TTMVx51) and panel (**d**) shows the novel *Gammatorquevirus* species (TTMDVx1–TTMDVx51).

According to the ICTV demarcation criteria, viruses belonging to the same genus but different species can be distinguished using an ORF1 nucleotide pairwise identity cutoff of 69%. In Fig. 3(a), we plot the pairwise ORF1 identities (calculated with SDT v1.2) between each newly discovered isolate and representative reference strains for the three genera. In all three genera, the vast majority of identity values lie below 69%, demonstrating that most isolate – reference comparisons fall into different species rather than the same species. For example, in *Alphatorquevirus* (blue), the pairwise identities range from ~50.7% to 98.1%, with a median of 55.8% (interquartile range 54.6%–57.6%). In *Betatorquevirus* (dark blue), values span ~52.1%–86.6% (median 56.8%, interquartile range (IQR) 55.8%–57.9%), and in *Gammatorquevirus* (green), they span ~52.7%–100% (median 58.9%, IQR 57.8%–60.2%). Because most points fall below the 69% cutoff line (dashed red), it is clear that there is extensive genetic diversity within each genus and that only a small fraction of comparisons exceed the species‐level threshold.

Based on pairwise nucleotide‐identity analysis, all 49 isolates assigned to the genus *Alphatorquevirus* were clustered into 15 novel species (comprising 25 isolates) and 10 established species (comprising the remaining 24 isolates) (Fig. 3b, Table S3). The 15 newly defined species have been named TTVx1 through TTVx15. Three of these species (TTVx1, TTVx14 and TTVx15) each encompass exactly three isolates: TTVx1 unites HTTVa_002, HTTVa_003 and HTTVa_026; TTVx14 brings together HTTVa_041, HTTVa_047 and HTTVa_048; and TTVx15 comprises HTTVa_042, HTTVa_045 and HTTVa_049. Four additional species are represented by two isolates apiece (TTVx2 by HTTVa_004 and HTTVa_030, TTVx3 by HTTVa_005 and HTTVa_039, TTVx5 by HTTVa_007 and HTTVa_020 and TTVx10 by HTTVa_029 and HTTVa_046). The remaining eight species (TTVx4, TTVx6, TTVx7, TTVx8, TTVx9, TTVx11, TTVx12 and TTVx13) are each defined by a single isolate (respectively, HTTVa_006, HTTVa_010, HTTVa_011, HTTVa_015, HTTVa_018, HTTVa_032, HTTVa_036 and HTTVa_040) (Table S3). The rest of 24 isolates belong to 10 established species (TTV1, TTV3, TTV6, TTV13, TTV17, TTV19, TTV20, TTV21, TTV24 and TTV31). (Table S4). Several of these established species each recruited multiple isolates (for example, TTV1 and TTV3 each include three new strains), whereas others contained only a single newly sequenced genome. This confirms that, in addition to the 15 novel species delineated above, a substantial proportion of our isolates fall within known *Alphatorquevirus* taxa.

**Fig. 4.**
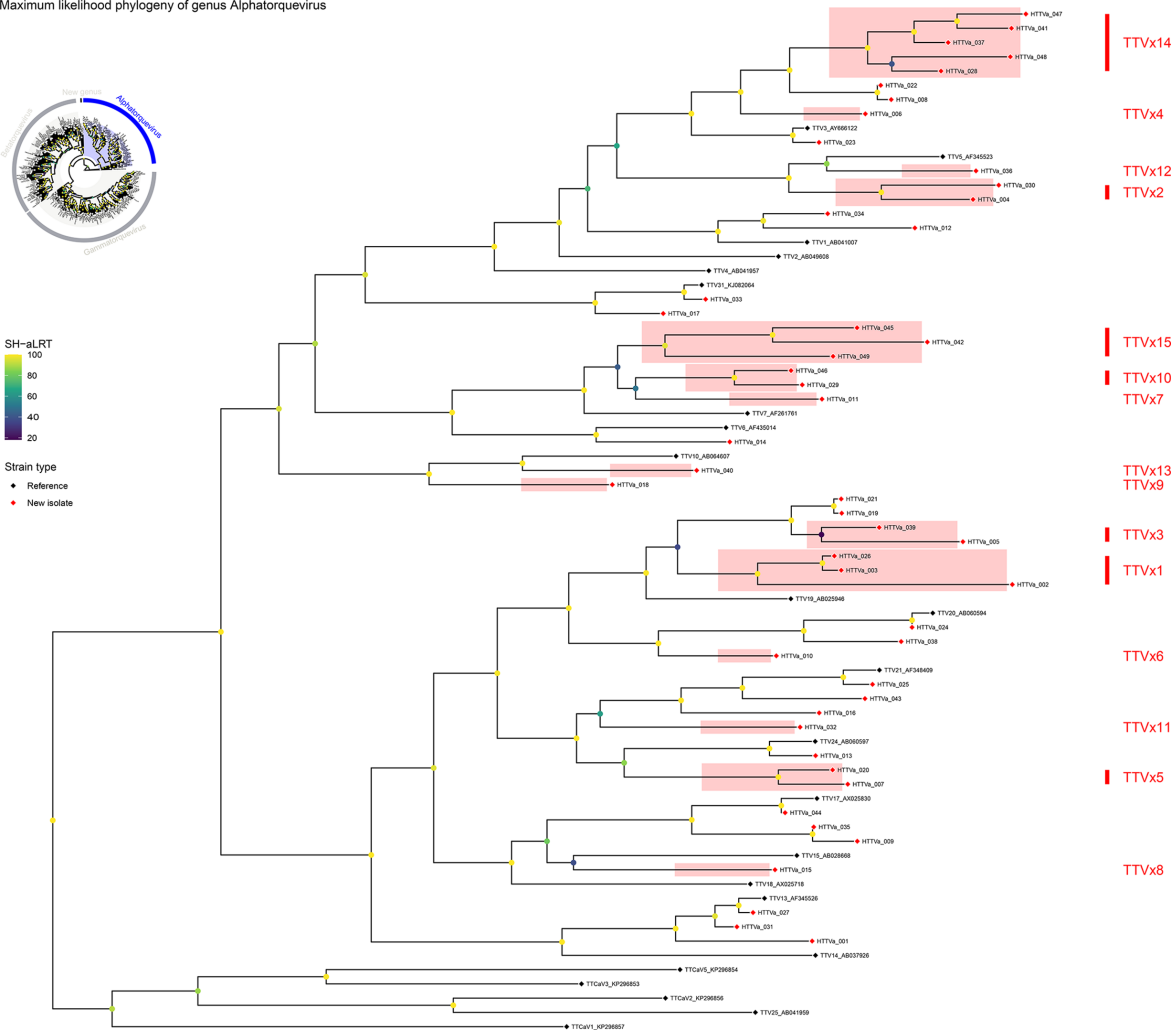
Maximum‐likelihood phylogeny of genus *Alphatorquevirus* (subset of Fig. 1). The tree includes only representative reference sequences and all newly identified HTTVa isolates (HTTVa_001–HTTVa_049). It was inferred in IQ‐TREE v2 under the LG+F+G4 model with 1,000 SH-aLRT replicates; branch labels indicate SH-aLRT support (%) on an emerald gradient. Tip symbols distinguish ‘Reference’ (black) versus ‘New isolate’ (red). Coloured box labels (TTVx1–TTVx15) mark each of the 15 novel species discovered by ORF1 pairwise identity (cutoff 69%).

Most newly proposed species segregate as discrete clades in the ORF1 amino acid tree (Fig. 4), validating our nucleotide‐based thresholds. For example, the reference strain TTV1 (AB041007) clusters with HTTVa_012 and HTTVa_034 – all three group exclusively and form the TTV1 clade – and the two novel isolates HTTVa_004 and HTTVa_030 coalesce into a single, well‐supported branch corresponding to our new species TTVx2. In contrast, two of our ‘novel’ species – TTVx14 (HTTVa_041, HTTVa_047 and HTTVa_048) and TTVx4 (HTTVa_006) – do not form independent lineages but instead nest within the established TTV3 clade (which also contains HTTVa_008, HTTVa_022, HTTVa_023, HTTVa_028 and reference TTV3_AY666122). This discordance indicates that, although TTVx14 and TTVx4 isolates fall below the 69% ORF1‐nucleotide identity cutoff relative to TTV3, their ORF1 amino acid sequences remain sufficiently similar to TTV3 members to group together phylogenetically. In other words, these isolates share greater protein‐level conservation with TTV3 than their nucleotide divergence alone would suggest, highlighting the fine line between intra‐ and interspecies variation in *Alphatorquevirus*.

### Novel species identified in the genus *Betatorquevirus*

Based on pairwise nucleotide‐identity analysis, all 62 isolates assigned to the genus *Betatorquevirus* were clustered into 51 novel species (comprising 56 isolates) and 4 established species (comprising the remaining 6 isolates). The 51 newly defined species have been named TTMVx1 through TTMVx51 (Fig. 3c). One of these species – TTMVx23 – comprises exactly three isolates (HTTVb_026, HTTVb_027 and HTTVb_061). Two additional species each contain exactly two isolates: TTMVx20 unites HTTVb_023 and HTTVb_048, and TTMVx25 brings together HTTVb_029 and HTTVb_058. Each of the remaining 48 species is represented by a single isolate: TTMVx1 by HTTVb_001, TTMVx2 by HTTVb_002, TTMVx3 by HTTVb_003, TTMVx4 by HTTVb_004, TTMVx5 by HTTVb_005, TTMVx6 by HTTVb_006 and HTTVb_057, TTMVx7 by HTTVb_007, TTMVx8 by HTTVb_008, TTMVx9 by HTTVb_009, TTMVx10 by HTTVb_010, TTMVx11 by HTTVb_012, TTMVx12 by HTTVb_013, TTMVx13 by HTTVb_014, TTMVx14 by HTTVb_015, TTMVx15 by HTTVb_016, TTMVx16 by HTTVb_018, TTMVx17 by HTTVb_020, TTMVx18 by HTTVb_021, TTMVx19 by HTTVb_022, TTMVx21 by HTTVb_024, TTMVx22 by HTTVb_025, TTMVx24 by HTTVb_028, TTMVx26 by HTTVb_030, TTMVx27 by HTTVb_031, TTMVx28 by HTTVb_032, TTMVx29 by HTTVb_033, TTMVx30 by HTTVb_034, TTMVx31 by HTTVb_035, TTMVx32 by HTTVb_036, TTMVx33 by HTTVb_037, TTMVx34 by HTTVb_039, TTMVx35 by HTTVb_040, TTMVx36 by HTTVb_041, TTMVx37 by HTTVb_042, TTMVx38 by HTTVb_043, TTMVx39 by HTTVb_044, TTMVx40 by HTTVb_045, TTMVx41 by HTTVb_046, TTMVx42 by HTTVb_047, TTMVx43 by HTTVb_049, TTMVx44 by HTTVb_050, TTMVx45 by HTTVb_051, TTMVx46 by HTTVb_052, TTMVx47 by HTTVb_054, TTMVx48 by HTTVb_055, TTMVx49 by HTTVb_056, TTMVx50 by HTTVb_059 and TTMVx51 by HTTVb_060 (Table S3). In every case, pairwise nucleotide identity between any two isolates assigned to the same TTMVx species exceeded 69%, whereas identities between isolates of different species fell below that threshold (Fig. 3a). The remaining six newly identified isolates were assigned to four previously established species: TTMV1 (HTTVb_011 and HTTVb_038), TTMV6 (HTTVb_053), TTMV26 (HTTVb_017 and HTTVb_019) and TTMV32 (HTTVb_062) (Table S4).

**Fig. 5.**
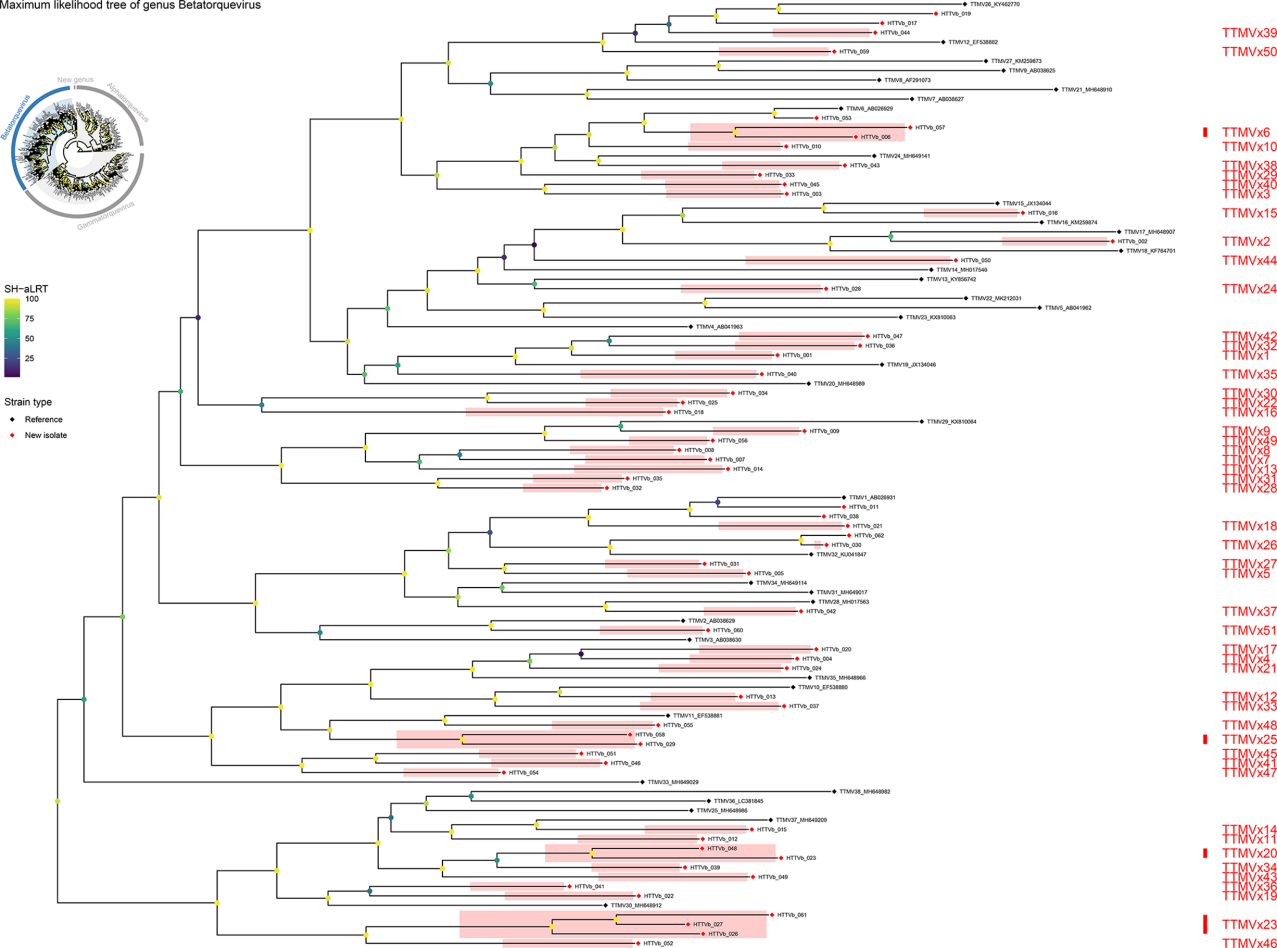
Maximum‐likelihood phylogeny of genus *Betatorquevirus* (subset of Fig. 1). The tree includes only representative reference sequences and all newly identified HTTVb isolates (HTTVb_001–HTTVb_062). It was inferred in IQ‐TREE v2 under the LG+F+G4 model with 1,000 SH-aLRT replicates; branch labels indicate SH-aLRT support (%) on an emerald gradient. Tip symbols distinguish ‘Reference’ (black) versus ‘New isolate’ (red). Coloured box labels (TTMVx1–TTMVx51) mark each of the fifteen novel species discovered by ORF1 pairwise identity (cutoff 69%).

In *Betatorquevirus*, every novel species defined by ORF1 nucleotide pairwise identity also forms its own distinct clade in the ORF1 amino acid phylogeny (Fig. 5). This perfect correspondence between nucleotide‐based grouping and protein‐level branching demonstrates that our 69% identity cutoff yields taxa that are both genetically and evolutionarily coherent. In other words, each TTMVx species appears as an isolated cluster, with strong SH‐aLRT support separating it from all other *Betatorquevirus* lineages. Thus, the maximum likelihood (ML) tree provides independent validation of our pairwise‐identity‐based species assignments.

### Novel species identified in the genus *Gammatorquevirus*

Based on pairwise nucleotide‐identity analysis, all 104 isolates assigned to the genus *Gammatorquevirus* were clustered into 51 novel species (comprising 75 isolates) and 11 established species (comprising the remaining 29 isolates). The 51 newly defined species have been named TTMDVx1 through TTMDVx51 (Fig. 3d). Among these novel species, TTMDVx1 unites four isolates (HTTVg_002, HTTVg_025, HTTVg_046 and HTTVg_102), TTMDVx2 groups two isolates (HTTVg_003 and HTTVg_063) and TTMDVx9 brings together three isolates (HTTVg_013, HTTVg_044 and HTTVg_069). TTMDVx7 is defined by five isolates (HTTVg_010, HTTVg_028, HTTVg_035, HTTVg_078 and HTTVg_079), while TTMDVx12 contains four isolates (HTTVg_021, HTTVg_026, HTTVg_042 and HTTVg_082). Each of the remaining 46 novel species is represented by a single isolate (for example, TTMDVx3 by HTTVg_004, TTMDVx4 by HTTVg_005, TTMDVx5 by HTTVg_006, TTMDVx6 by HTTVg_009, TTMDVx8 by HTTVg_011, TTMDVx10 by HTTVg_015, TTMDVx11 by HTTVg_020 and so on through TTMDVx51 by HTTVg_103) (Table S3). In every case, pairwise nucleotide identities among isolates within a given TTMDVx species exceeded the 69% threshold, whereas identities between isolates of different species fell below 69% (Fig. 3a).

Similarly, in *Gammatorquevirus*, the remaining isolates fell into established TTMDV species – multiple isolates mapped to TTMDV1, TTMDV2, TTMDV6, TTMDV8, TTMDV9, TTMDV10, TTMDV11, TTMDV12, TTMDV13 and TTMDV14 – based on their ORF1 nucleotide identities with reference sequences (Table S4).

**Fig. 6.**
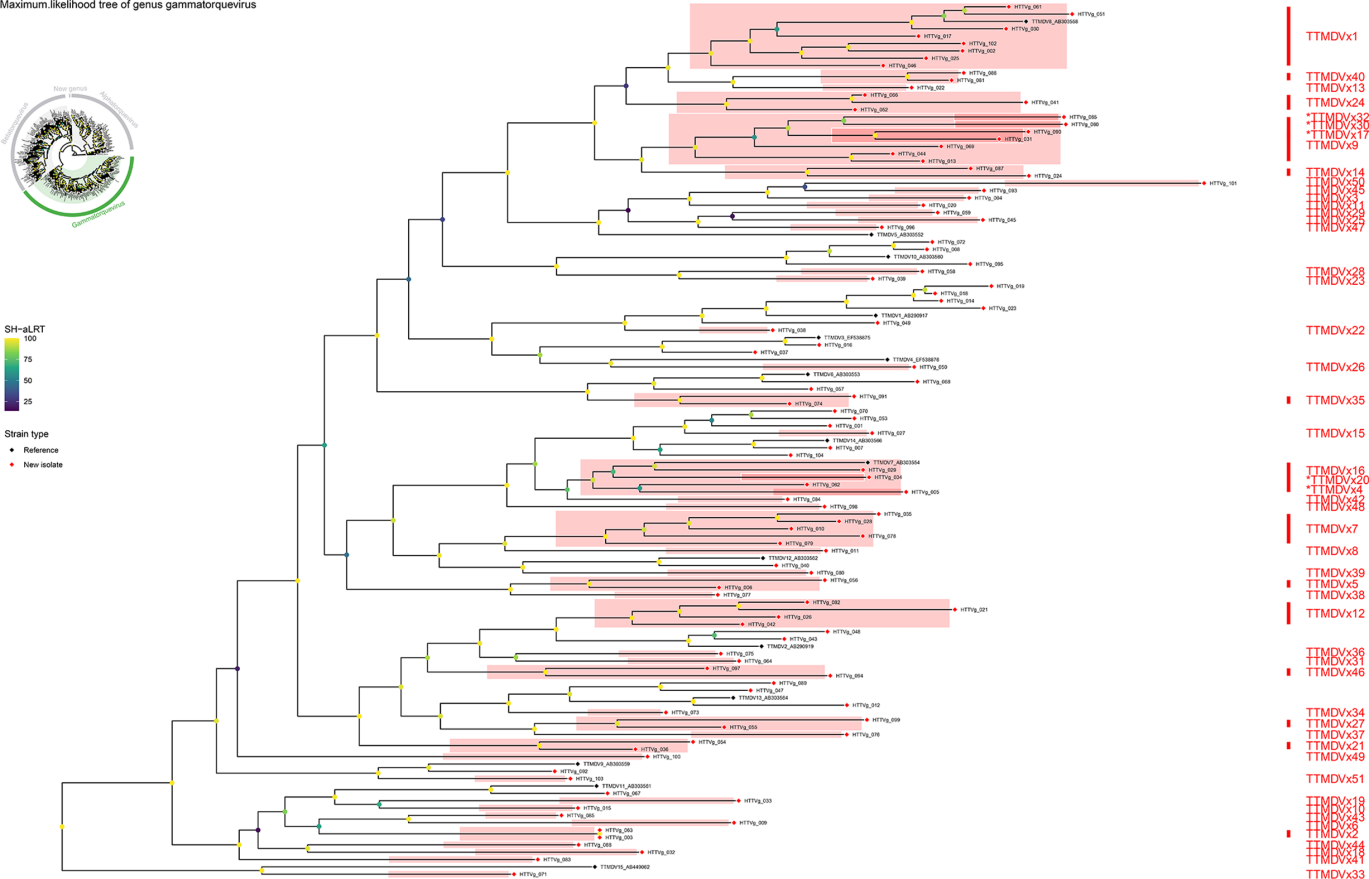
Maximum‐likelihood phylogeny of genus *Gammatorquevirus* (subset of Fig. 1). The tree includes only representative reference sequences and all newly identified HTTVb isolates (HTTVg_001–HTTVg_104). It was inferred in IQ‐TREE v2 under the LG+F+G4 model with 1,000 SH-aLRT replicates; branch labels indicate SH-aLRT support (%) on an emerald gradient. Tip symbols distinguish ‘Reference’ (black) versus ‘New isolate’ (red). Coloured box labels (TTMDVx1–TTMDVx51) mark each of the 15 novel species discovered by ORF1 pairwise identity (cutoff 69%).

As observed in *Alphatorquevirus*, most novel TTMDVx species defined by ORF1 nucleotide pairwise identity also appear as independent clades in the ORF1 amino acid phylogeny (Fig. 6). However, several exceptions highlight residual amino‐acid conservation that blurs strict nucleotide‐cutoff boundaries. For example, the newly proposed TTMDVx1 group (HTTVg_002, HTTVg_025, HTTVg_046 and HTTVg_102) does not form its own lineage but instead nests within the established TTMDV8 clade (HTTVg_017, HTTVg_030, HTTVg_051 and HTTVg_061). Likewise, four novel species – TTMDVx9 (HTTVg_013 and HTTVg_044), TTMDVx17 (HTTVg_031), TTMDVx30 (HTTVg_060) and TTMDVx32 (HTTVg_065) – cluster together as a single branch rather than splitting into discrete groups. In addition, three novel species – TTMDVx4 (HTTVg_005), TTMDVx16 (HTTVg_029) and TTMDVx20 (HTTVg_034) – coalesce with the previously recognized TTMDV4 reference (6PoSMA/EF538876). These patterns indicate that, although these isolates fall below the 69% ORF1‐nucleotide identity threshold relative to their nearest neighbours, they retain sufficient protein‐level similarity to group them with existing TTMDV lineages. Thus, the ML phylogeny largely corroborates our nucleotide‐based species assignments while revealing instances of overlapping amino acid conservation in *Gammatorquevirus*.

### Key motif conserved in novel species of anellovirus capsid protein

#### Conserved capsid fold across genera

**Fig. 7.**
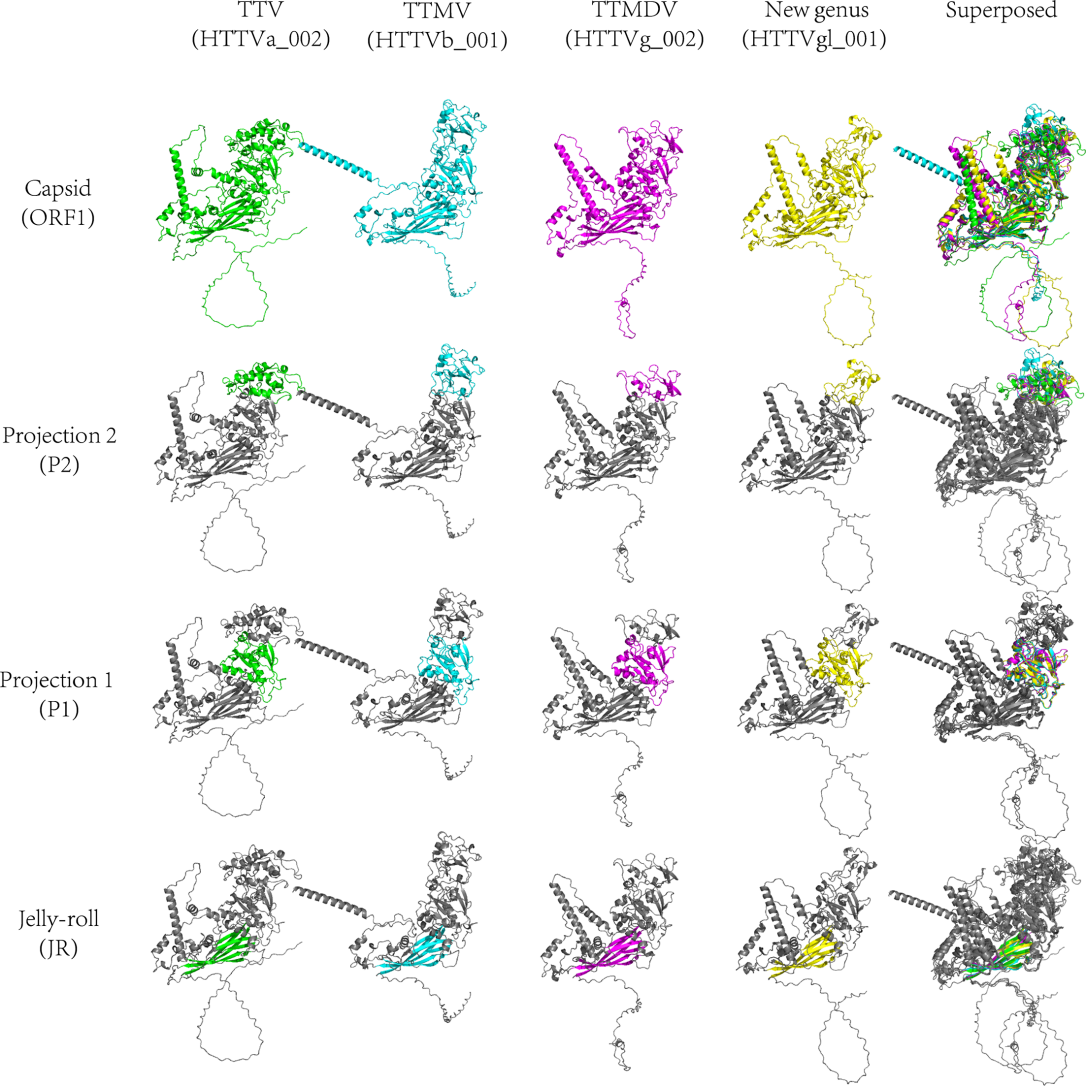
AlphaFold-predicted structures of anellovirus ORF1 (capsid) highlight conserved JR, P1 and P2 motifs across genera. From left to right: TTV (HTTVa_002, green), TTMV (HTTVb_001, cyan), TTMDV (HTTVg_002, magenta), NG (HTTVgl_001, yellow) and superposed. Top row: full ORF1 models (disordered N-terminal tails retained for context). Second to fourth rows: the same models greyed with the P2, P1 or JR region coloured to illustrate boundaries and relative sizes. The superposed column shows the overlap of all four models, emphasizing the conserved JR *β*-sandwich core and comparable P1/P2 placements. Region lengths (JR, **p1, p2**) were quantified from the AlphaFold models as the number of residues assigned to each region (see Methods).

AlphaFold3 modelling of ORF1 from four representatives – HTTVa_002 (TTV, green), HTTVb_001 (TTMV, cyan), HTTVg_002 (TTMDV, magenta) and HTTVgl_001 (proposed NG, yellow) – reveals a shared capsid architecture (Fig. 7). All models exhibit the canonical JR *β*-sandwich core flanked by two outward projection domains (P1 and P2), consistent with anellovirus capsid organization. Superposition after JR-core alignment shows near-identical packing of the JR core across lineages, with genus-specific differences largely confined to surface-exposed loops within P1/P2. As expected for these proteins, N-terminal low-complexity segments are poorly ordered and of low confidence and were not used for quantitative comparisons.

### Residue-level conservation of the JR *β*-sandwich (*β*-strands B–I)

MSA of representative ORF1 capsids – including three references (species TTV21/AF348409, TTMV15/JX134044 and TTMDV3/EF538875) and newly identified isolates – shows strong conservation within the JR core (Fig. S2). In particular, residues mapping to the eight *β*-strands (B, C, D, E, F, G, H and I) that compose the two *β*-sheets of the JR are topologically aligned and sequence-constrained across all genera examined (Fig. 2a, b). By contrast, P1/P2 loops that project from the JR framework display higher sequence diversity and length variability, consistent with their predicted surface exposure.

### Motif lengths corroborate structural conservation and genus-specific patterns

Quantitative motif lengths derived from the structural/sequence mapping (JR, P1 and P2) further support these observations (Fig. S3; motif length summary). JR is tightly conserved across both newly identified and reference sequences, with medians clustered at ~186 aa in all three established genera (TTV, TTMV and TTMDV). For example, TTV (median 186 aa in new isolates; 186 aa in references), TTMV (186/186 aa), and TTMDV (186/186 aa) all show narrow IQRs around this value, indicating strong size constraint of the *β*-sandwich core. The NG shows the same JR length (median 186 aa; *n*=2), consistent with a conserved core fold. P1 displays modest genus-specific dispersion but broad overlap between new and reference sets. Median lengths are ~167 aa (TTV) and ~163 aa (TTMV/TTMDV), with NG ~167.5 aa (*n*=2) – again within the interquartile ranges of established genera. P2 shows the largest inter-genus shift, following a TTV>TTMV>TTMDV gradient in median length (~151, ~125 and ~89–90 aa, respectively). Notably, NG P2 (~90 aa; *n*=2) aligns with the shorter TTMDV-like state, suggesting that while the JR core is invariant, surface projections capture lineage-specific differences.

Collectively, the structural superposition, *β*-strand-level alignment of the JR (B–I) and motif-length statistics converge on the same conclusion: the proposed NG retains the canonical anellovirus capsid core and motif organization. The JR *β*-sandwich is strongly conserved in both topology and length, whereas P1/P2 tolerate genus-specific loop variation without systematic expansion or truncation relative to references. These findings provide functional/structural context for the phylogenetic placement of the new lineage, indicating deep sequence divergence with preservation of the capsid’s key architectural motifs.

## Discussion

Anelloviruses are characterized by extremely high prevalence with relatively uniform distribution worldwide and a high level of genomic heterogeneity. Since the first HTTVs were discovered in a hepatitis patient in 1997 [22], a large number of HTTV isolates have been identified. Currently, 8 genera including 3 main genera of *Alphatorquevirus* (26 validated species, including 6 species infect animals), *Betatorquevirus* (38 species) and *Gammatorquevirus* (15 species) infect human [4].

Recent family-wide analyses and structural modelling indicate that ORF1 encodes the principal capsid protein that adopts a SJR *β*-sandwich core, thereby linking anelloviruses to other eukaryotic ssDNA viruses, with lineage-specific projection domains inserted into the JR – often between *β*-strands H and I [14]. cryo-electron microscopy and virus-like particles (VLP) studies further reveal features consistent with immune evasion on the capsid surface [38]. In our dataset, AlphaFold3-guided annotation showed that the JR *β*-strands B–I are conserved across *Alphatorquevirus*, *Betatorquevirus* and *Gammatorquevirus*, whereas P1/P2 projections vary in length and loop composition; notably, the NG resembles TTMDV-like capsids in having a shorter P2, while retaining an invariant JR core. These observations provide a functional/structural framework that complements our phylogenetic placements.

Our large‐scale survey of HTTVs from published gut metagenomes has uncovered an unexpectedly rich array of novel species, nearly doubling the known species count in the three major human‐infecting genera. By applying a 69% ORF1 nucleotide‐identity threshold in line with ICTV guidelines, we identified 117 previously unrecognized species – 15 in *Alphatorquevirus*, 51 in *Betatorquevirus* and 51 in *Gammatorquevirus –* as well as two isolates (HTTVgl_001 and HTTVgl_002) that form a deeply branching, distinct lineage sufficiently divergent to merit provisional recognition as a separate genus. In almost every instance, these sequence‐based species assignments map exactly onto well‐supported, monophyletic clades in maximum‐likelihood trees of ORF1 amino acid alignments. A small number of cases where pairwise‐based clusters split or merge differently serve as useful reminders that viral genomes continually diversify and that fixed numerical cutoffs may occasionally yield borderline groupings.

The dramatic expansion of anellovirus species richness raises two pressing challenges for taxonomy. First, the flood of new lineages makes it impractical to assign formal species epithets via conventional ICTV processes for each small cluster; therefore, we have provisionally labelled new groups ‘TTVx’, ‘TTMVx’ and ‘TTMDVx’ purely as temporary identifiers to track genetic clusters until more detailed phenotypic or epidemiological data can support official naming. Second, as more metagenomic studies across diverse populations produce thousands of novel anellovirus genomes, the field must develop scalable conventions – such as automated clustering thresholds combined with lineage codes – to keep nomenclature both consistent and informative without becoming unwieldy.

Beyond taxonomy, our previous findings underscore the value of anelloviruses as clinical biomarkers of host immunity [13,39, 40]. Numerous studies have shown that anellovirus abundance rises under immunosuppression and declines upon immune reconstitution. For example, in simian immunodeficiency virus-infected macaques, large expansions of plasma anelloviruses closely track CD4+ T‐cell loss, and in HIV‐1 patients, anellovirus titres remain elevated until long‐term antiretroviral treatment restores immune function [41,42]. Our broadened catalogue now provides more precise sequence references for qPCR assays or metagenomic classifiers that can distinguish among newly discovered species – potentially improving sensitivity and specificity when monitoring patients’ immune status in transplantation, oncology or chronic infection settings.

Interest in anelloviruses as delivery scaffolds has increased [43,44]. However, broad seroreactivity to anellovirus peptides has been documented using large phage-display libraries (VirScan/AnelloScan), with antibody responses detected to multiple ORFs – including ORF1 and ORF2 – across individuals and cohorts [45]. Combined with structural evidence that variable P1/P2 loops form the outward projection domain [14,38], these data suggest that pre-existing humoral immunity is likely common and may be genus- and loop-specific. Accordingly, we hypothesized that rational capsid selection might focus on lineages with divergent P1/P2 loops and minimal expected seroprevalence in target populations, but neutralization and cross-reactivity assays (e.g. VLPs from synthetic genomes [43] and curated peptide/epitope panels [45]) are required to evaluate susceptibility to existing antibodies. Our structure-guided motif maps (JR B–I; P1/P2) provide a starting point for such epitope-aware designs, but immunological data are still needed before any claims about reduced pre-existing immunity can be made.

Anelloviruses are likewise gaining traction as viral vectors. Their noncytopathic nature, broad tropism and ability to establish persistent, low‐level infections make them appealing scaffolds for gene delivery. One recent, non-peer-reviewed study demonstrated that engineered anellovirus backbones can deliver reporter genes to multiple tissues in animal models [44], suggesting a platform for long‐term expression of therapeutic payloads. Consistent with these developments, recombinant production of human anelloviruses from synthetic genomes has been reported [43], and a gene-delivery platform based on a commensal human anellovirus has shown transduction in multiple tissue types [44]. By mining our newly identified species, researchers might select capsid – ORF1 scaffolds with minimal preexisting immunity in target human cohorts, thereby reducing neutralization and enhancing transduction efficiency. Nevertheless, given the widespread antibody reactivity to anelloviruses [45], vector performance depends on empirical neutralization profiles.

The discovery of two isolates (HTTVgl_001 and HTTVgl_002) comprising a novel genus highlights that even well‐studied niches like the human gut still conceal deeply divergent anelloviruses. These isolates share only 55.8% ORF1 identity with any known HTTV, placing them outside existing human genera yet within the larger *Anelloviridae* clade that includes *Alphatorquevirus, Betatorquevirus*, *Gammatorquevirus*, *Epsilontorquevirus* and *Zetatorquevirus*. Their identification suggests that additional, highly divergent lineages await discovery – perhaps in under‐sampled populations, low‐abundance reservoirs or anatomical compartments not yet thoroughly sequenced. From an evolutionary perspective, our observations are consistent with the view that anelloviruses derive from a circovirus-like SJR capsid ancestor, with diversification largely mediated by augmentation of the projection domain on an ORF1 scaffold [14]; our NG conforms to this pattern by maintaining the conserved JR core while adopting a distinct projection architecture. Finally, legacy reports of putative Rep motifs in ORF1 [46] are increasingly difficult to reconcile with structural and biochemical data that favour a primary capsid role [14,38]; our analyses also did not detect canonical Rep features in the newly assembled ORF1 sequences.

## Conclusion

By combining a rigorous, ICTV‐aligned identity threshold with robust phylogenetic analysis, we have dramatically expanded the known landscape of human anellovirus diversity. Our work not only nearly doubles the number of recognized HTTV species but also introduces provisional identifiers for tracking future discoveries and reports a novel genus. These findings have immediate practical implications: they provide the sequence resources needed to develop next‐generation assays for immune monitoring and lay the groundwork for exploiting anelloviruses as gene‐delivery vectors. In addition, our structure-guided analysis of ORF1 – highlighting a conserved JR core (*β*-strands B–I) with lineage-specific projection domains – places the new isolates in a biologically coherent framework consistent with recent family-wide studies [14,38]. Given the broad antibody reactivity to anellovirus peptides described in large cohort screens [45], we explicitly treat vector implications as hypotheses: candidate capsids from divergent lineages can now be prioritized, but their susceptibility to pre-existing immunity must be determined in neutralization and cross-reactivity assays (e.g. VLPs from synthetic genomes [43]). As metagenomic sequencing proliferates, the anellovirus field must adopt scalable, transparent naming conventions and pair genomic discovery with functional validation – including capsid structure, receptor usage and serology – to translate diversity into clinical and biotechnological advances [43,45].

## Abbreviations

HTTV: human torque teno virus
IQR: interquartile range
JR: jelly-roll
ML: maximum likelihood
MSA: multiple-sequence alignment
NG: new genus
PAE: predicted aligned error
pLDDT: predicted local distance difference test
SDT: sequence demarcation tool
SH‐aLRT: Shimodaira–Hasegawa approximate likelihood ratio test
SJR: single jelly-roll
TTMDV: torque teno midi virus
TTMV: torque teno mini virus
TTV: torque teno virus
VLPs: virus-like particles
